# Mental disorders may prevent, not cause, suicide

**DOI:** 10.1192/bjb.2024.50

**Published:** 2025-04

**Authors:** Annie Swanepoel, C. A. Soper

**Affiliations:** 1 North East London NHS Foundation Trust, UK; 2 Lisbon, Portugal

**Keywords:** Suicide, evolution, depression, addiction, self-harm

## Abstract

We challenge a prevalent belief that depression causes suicide and propose that certain symptoms of depression and other psychopathologies may function to *prevent* lethal self-injury. Theoretical and empirical evidence supports this position. As suicide posed an extreme fitness hazard throughout human evolution, our species evolved special-purpose psychological defences that continuously monitor and manage this danger. Last-ditch protections may present as diverse psychiatric phenomena. Mobilising in adolescence and adulthood in response to chronic distress, these usually stop suicidal thoughts from escalating into deadly actions. The theory is testable. We point to important implications for the clinical management of suicide and psychopathology.

Suicides are caused by depression, aren't they? Many people think so, and it is easy to see why. No doubt, mental illness and suicide connect somehow. Most people who die by suicide qualified for a psychiatric diagnosis at the time of death, and virtually all diagnostic categories carry an elevated suicide risk.^1^

## Mental disorders correlate with but rarely cause suicide

However, a closer look shows correlation confused with causation. Psychopathology – a risk factor for suicide – has been mistaken for an *explanatory* factor,^2^ and the mistake leaves much unexplained. Many suicides happen with no detectible mental illness, and the great majority of people with even the most serious psychiatric diagnoses do not take their own lives. Moreover, qualitative research into suicide seldom points to psychiatric problems as the main causal driver.^3^

The correlations don't fit the presumed causation anyway. Almost all of the association between mental illness and suicide relates to suicidal *thoughts*, not the *enacting* of those thoughts. Depression associates strongly with suicidal ideas, which many people experience, but only weakly with the much rarer progression of those ideas into firm plans and lethal actions.^4^ Moreover, the risk of suicide can be higher, not lower, when psychiatric symptoms start to ease.^5,6^ By this light, the idea that mental disorders cause suicide is almost certainly wrong.

## Suicide as a biological problem

There is a better way to explain the link between mental illness and suicide. Diverse symptoms of psychopathology may be expressions of evolved defences against suicide: they may function to *prevent* people from taking their own lives.

To understand why, we have to consider how suicide has shaped the evolution of the human psyche. A new theory of suicide's evolutionary origins, the ‘pain–brain’ theory, does just that.^7^ The main idea is that suicide poses a serious problem for an animal that has both the motivation and the means to do it.

The *motivation* lies in a driver that all of us experience at some stage: pain, especially psychological pain or ‘psychache’.^8^ Pain is designed not to be tolerated: it demands action to relieve or end it.

The *means* for suicide start with a brain so intelligent that it can mentalise self-induced death as a permanent end to pain. Virtually all of us develop this deadly intellectual capability, usually during the teen years. And, presumably, it was ever thus: *Homo sapiens* has lived with this very human problem ever since our ancestors became smart enough to think the thought. An occupational hazard of extreme sapience, suicide is an endemic threat to survival in our cognitive niche in the same way that, say, orangutans in the forest canopy face the everyday danger of a potentially fatal fall, and polar bears on the sea ice are at perpetual risk of hypothermia.

In sum, virtually all mature humans are at risk, as we are likely both to suffer pain and to know that suicidal escape is available.

## Evolved defences against suicide

The key question is this: why is suicide not more common? The answer is that the process of evolution has armed us against our special hazard. Orangutans, polar bears and humans thrive in what would otherwise be unsurvivable conditions because they are equipped with specialist, evolved solutions, fine-tuned over countless generations of natural selection. Thanks to these adaptations – and although accidents happen – orangutans rarely fall, polar bears rarely freeze and humans rarely kill themselves.

Multiple barriers protect humans from suicide, like ranks of defenders on a battlefield. The front line tries to stop suicidal thoughts from entering our heads in the first place. It keeps most of us, most of the time, fairly happy, fairly hopeful and able to bounce back from adversities. Some buffers use cultural inputs, such as believing that suicide is against God's will.

Our last-ditch defences are drastic measures for dangerous times.^1^ When life becomes so painful for so long that the prospect of ending it all could start to appeal, protections are triggered that seek to block any such thoughts from progressing into lethal action. These safeguards are developmentally scheduled to meet the emergence of the suicide hazard (i.e. from adolescence onwards), and they mobilise in response to chronic psychache. By interfering with normal cognitive and motivational systems, they usually make a fatal suicide attempt difficult and/or unnecessary. These shields are costly, often debilitating, but they are less costly than the terminal alternative.

## Final defences may display as psychiatric symptoms

These emergency anti-suicide protections may display as diverse symptoms of adolescent- and adult-pattern psychosomatic psychopathology. Negative symptoms sap the motivation for action and impair the ability to plan and organise. This could explain why depression correlates with suicidal ideas and not with the acting out of those ideas, and why there can be greater danger of suicide as symptoms lift.^5,6^ Non-suicidal self-injury, often comorbid with depression, may serve as a sublethal way to regulate potentially suicidogenic distress.^9^ Substance and behavioural addictions may similarly be understood as self-medication – coping mechanisms that, despite bringing new dangers, usually keep a potentially suicidal individual alive to see another day. Psychotic hallucinations may make self-destructive thoughts easier to resist by externalising them.^10^

Depending on age, sex, personality and other variables, different individuals may experience different blends of these and other protective measures, forming heterogeneous syndromes. By this approach, many commonplace psychiatric symptoms may be understood as evolved anti-suicide responses.

## A testable and useful theory

This theory won't explain all non-organic psychiatric diagnoses. We note two provisos. First, it doesn't account for early childhood disorders: evolved defences against suicide should mobilise only rarely before the developmental stage when suicidal behaviour typically first emerges, i.e. seldom before the early teen years. Second, it does not distinguish between different DSM-style diagnoses: being artificial constructs rather than ‘natural’ entities, diagnostic categories are unlikely targets for any evolutionary biological explanation. Nonetheless, the model tallies with a swathe of pan-diagnostic epidemiological patterns in adolescence and adulthood that are otherwise difficult to explain: extreme comorbidity, shared age at earliest onset, non-specificity of treatments and mutual aetiology, for example.

We have yet to see persuasive counterevidence and, importantly, this is not because the theory is unfalsifiable. Several testable predictions by which the theory could be affirmed or refuted are set out in the literature.^1^ Here are two examples. First, if it is true that psychache drives thoughts of ending the pain by suicide, thoughts that are stopped from being executed by evolved mechanisms such as a loss of energy and motivation, then there should be evidence that suicidal thoughts often occur early in psychopathology rather than being a late symptom. This sequencing would support the pain–brain model while contradicting the opposing idea that psychopathology causes suicide. Second, the theory points to chronic pain conditions as a cross-diagnostic risk factor: as pain of any kind can be unbearable, and if certain cross-diagnostic symptoms function to prevent suicidal thoughts being enacted, then chronic physiological pain, not just psychache, should be a risk factor for both suicidal thoughts and psychopathology.

Clinicians may find the theory helpful. It explains why suicide is opaque to usefully accurate risk assessment by any known method – why our ability to predict suicidal outcomes is little better than chance, and why ≥95% of people assessed as supposedly ‘high risk’ do not take their own lives.^11^ As the brain has an evolutionary head-start over our modern risk-assessment tools, any predictors detectible to us now have already been absorbed, actioned and thereby emptied of predictive value. Completed suicides are the near-random residue left after the psyche has done the best it practicably can with the information available.

It may also help to recognise that although none of us is immune, the evolved mind is expert at avoiding self-destruction. Humans needn't perpetually worry about suicide for the same reasons that orangutans and polar bears can get on with their lives without being bothered by (respectively) heights and the cold: we, like them, are biologically equipped to handle the special hazard that comes with our evolutionary habitat. Equipped, that is, for the habitat that prevailed in our evolutionary past: quick and easy means of lethal self-injury probably did not feature in our ancestral environment and are an Achilles’ heel today. Keeping guns, pesticides and other death-traps out of harm's way is a sensible precaution: it buys time for the psyche's defences to stand down from potentially deadly crises.

As clinicians, if we try not to let our anxiety about suicide get in the way, and if we keep a sense of proportion as to the realistic level of risk, we can focus instead on trying to understand and ease patients’ underlying distress – which is usually not about suicide.^11,12^ As for mental illness, to be clear, we are not saying that psychopathology should be left untreated. Of course, pharmacotherapy has an important role. We are saying that medication cannot be the be-all and end-all. If this theory is broadly right, mental disorders often stem from a common root: psychache. We need to look beyond the diagnosis to the underlying pain and try to alleviate that as well. In this respect, we are no different to any other medical specialty: symptomatic treatment must not be conflated with curative treatment, but they may be used together.

## A new direction for research in mental health

Science has barely begun to acknowledge the existence of anti-suicide adaptations, let alone ascertain how they work. However, the evidence, empirical and theoretical, suggests that psychiatric symptoms may play a vitally protective part in many cases. We acknowledge that the pain–brain theory about the link between suicide and psychopathology does not explain all mental illness. However, we think that it is worth further consideration, as it asks novel questions, suggests testable avenues for research and has profound implications for clinical practice and risk assessments.

## About the authors

**Annie Swanepoel**, PhD, MRCPsych, CCT, is a consultant child and adolescent psychiatrist with North East London NHS Foundation Trust, UK. **C. A. Soper**, PhD, MBA, MA, is an independent researcher and psychotherapist in private practice in Lisbon, Portugal.
